# The role of p21-activated kinase in the initiation of atherosclerosis

**DOI:** 10.1186/1471-2261-12-55

**Published:** 2012-07-23

**Authors:** K A Jhaveri, P Debnath, J Chernoff, J Sanders, M A Schwartz

**Affiliations:** 1 Department of Microbiology, University of Virginia, Cardiovascular Research Center, Charlottesville, Virginia, USA; 2 Division of Oncology Research, Fox Chase Cancer Center, Buckingham, Pennsylvania, USA; 3 Yale Cardiovascular Research Center, 300 George St., New Haven, CT 06511, USA

**Keywords:** Fluid shear stress, Endothelial cells, Vascular inflammation, Fibronectin

## Abstract

**Background:**

p21-activated kinase (PAK) has been implicated in the inflammatory activation of endothelial cells by disturbed fluid shear stress, which is the initiating stimulus in atherosclerosis. The study addresses whether PAK1 contributes to inflammatory marker expression in endothelial cells at atherosclerosis-susceptible regions of arteries in vivo.

**Method:**

Aortas from WT and PAK1^-/-^ C57BL/6J mice on a normal chow diet were fixed, dissected and processed for immunohistochemistry using a panel of inflammatory markers. We visualized and quantified staining in the endothelium at the greater and lesser curvatures of the arch of aorta, as atherosclerosis-resistant and susceptible regions, respectively.

**Results:**

Fibronectin, VCAM-1 and the activated RelA NF-κB subunit were localized to the lesser curvature and decreased in PAK1-/- mice. The activated RelB NF-κB subunit was also localized to the lesser curvature but was increased in PAK1-/- mice. Low levels of staining for ICAM-1 and the monocyte/macrophage marker Mac2 indicated that overall inflammation in this tissue was minimal.

**Conclusion:**

These data show that PAK1 has a significant pro-inflammatory function at atherosclerosis-prone sites in vivo. These effects are seen in young mice with very low levels of inflammation, suggesting that inflammatory activation of the endothelium is primarily biomechanical. Activation involves NF-κB, expression of leukocyte recruitment receptors and fibronectin deposition. These results support and extend in vitro studies demonstrating that PAK contributes to activation of inflammatory pathways in endothelial cells by fluid shear stress.

## Background

Atherosclerosis occurs mainly at regions of vessel curvature, branch points and bifurcations. These regions are distinguished by blood flow patterns with features such as low shear stress, flow separation and reattachment, and flow reversal during the cardiac cycle (so-called oscillatory flow) [1,2]. Application of analogous flow patterns to endothelial cells (ECs) in vitro recapitulates many of the effects seen in vivo at atherosclerosis-prone sites, including inflammatory gene expression, increased permeability, leukocyte recruitment and cell turnover [3-5]. In vivo, these atherosclerosis-prone regions of arteries show mild chronic inflammation without plaque formation even in WT mice or other organisms that do not normally develop atherosclerosis [6]. Progression to atherosclerotic plaque requires additional risk factors such as hyperlipidemia or diabetes [2]. Taken together, these results suggest that biomechanical activation of the endothelium by disturbed flow is the initiating event that determines the spatial distribution of atherosclerotic plaques, whereas systemic risk factors contribute mainly to the progression of mild inflammation into atherosclerosis [7,8].

p21-activated kinases (PAKs) comprise a highly conserved family of serine/threonine protein kinases that are important effectors of Rac and Cdc42. PAKs have been implicated in proliferative signaling by growth factor receptor tyrosine kinases, in control of cell polarity and actin cytoskeletal organization in higher eukaryotic cells [9]. On the basis of biochemical and structural features, PAKs are classified into Group I, consisting of PAK1-3, and Group II, consisting of PAK4-6 [10,11]. Among group I PAKs, PAK1 and -2 are widely expressed, including in endothelial cells, whereas PAK3 is found largely in the brain. PAK substrates include components of the mitogen-activated protein (MAP) kinase pathway, cytoskeletal proteins such as filamin and Op18, regulators of cell survival, and the transcription factor nuclear factor (NF) – κB.

Previous work from our lab showed that in endothelial cells, application of fluid shear stress activates PAK, which subsequently controls junctional integrity and vascular permeability [12-14]. Moreover, PAK is a critical modulator of several inflammatory pathways, including NF-κB and JNK, such that PAK activity is required for flow-dependent activation of these pathways and induction of downstream genes. Furthermore, PAK activation in mouse arteries was observed specifically in atherosclerosis-prone sites and correlated with inflammatory gene expression, including vascular cell adhesion molecule-1 (VCAM-1). PAK inhibitors reduced NF-κB and JNK phosphorylation in ECs in response to fluid shear stress in vitro, and reduced vascular permeability in vivo, indicating its functional significance in these processes.

Remodeling of the subendothelial extracellular matrix (ECM) appears to be an important component of atherosclerotic progression [15]. Fibronectin localizes to the subendothelial ECM selectively in atherosclerosis-prone regions in vivo. In vitro, ECs on basement membrane proteins show reduced inflammatory activation in response to flow and other stimuli. By contrast, cells on fibronectin, an ECM protein associated with remodeling, injury and inflammation, respond strongly to the same stimuli. PAK showed enhanced activation by flow, oxidized LDL and MCP-1 in cells on fibronectin compared to basement membrane proteins, and this differential activation of PAK mediates the ECM-specific activation of NF-κB and JNK [13,14]. These effects form part of a positive feedback loop in which inflammatory pathways promote fibronectin gene expression and matrix assembly, which subsequently enhances inflammation. Genetic studies in mice and humans support a causal role for ECM remodeling in atherosclerosis [16-18].

Previous studies of PAK in these processes were based on the use of PAK inhibitors that are isoform nonspecific and may have other non-specific effects. Therefore, to further define the role of PAK in the biomechanical initiation of the atherosclerotic cascade, we examined inflammatory activation of the endothelium at atherosclerosis-prone sites in PAK-1 knockout mice. These studies suggest that PAK1 plays a significant role in initiation of atherosclerosis.

## Methods

### Cell culture

8-10 week old C57BL/6J mice were sacrificed, pressure perfused, and the thoracic aortas were isolated. The aorta was cut in 1 mm segments, which were placed on a bed of matrigel with enriched EBM-2 media (see below). Cells grow and migrate out into the matrigel bed over 3-6 days. On day 7, the tissue was removed and cells grown for an additional 3-6 days when they covered the bottom of the well. Cells were released from the gel with dispase, which was diluted in 3 cc of DMEM media (with D-valine) containing 15% heat inactivated FBS (“isolation medium”). Cells were isolated by low speed centrifugation and plated on 0.25% gelatin coated dishes in this medium to inhibit fibroblast growth. After day 7, cells were switched to EBM-2 media containing 10% FBS, 1% bovine brain extract, 60 μg/mL heparin (Sigma), 10 U/ml penicillin, and 10 μg/ml streptomycin and Growth factor kit from Lorna Biosciences (growth medium). Endothelial cell identity was tested by LDL uptake. Cell lysates were run on a 10% SDS-PAGE gel and probed for total PAK1-C19 antibody (recognizes both PAK1 and 2) at 1:1000 primary antibody dilution.

### Animals

PAK-/- C57Bl/6J mice were originally generated as described in Clapp et al., 2009. Wild type C57Bl/6J mice were purchased from Jackson Laboratories (Bar Harbour, Maine). We used male 10-12 weeks old PAK KO, PAK HZ, and wild type C57BL/6J mice for our study (n = 7 for PAK HZ and C57BL/6 and n = 6 for PAK KO). All mice were housed in temperature-controlled chambers and fed a normal chow diet. The Laboratory Animal Care and Use Committee of the Comparative Medicine Department at University of Virginia approved all animal procedures used in this study.

### Immunohistochemistry

Animals were sacrificed and pressure-perfused with cold 4% paraformaldehyde in 1X phosphate-buffered saline. The complete aortas were dissected, processed, and embedded in paraffin blocks. 5 μm longitudinal sections of the aorta were used for immunohistochemistry (IHC). Following microwave antigen retrieval with antigen unmasking solution (Vector Labs), sections were stained for: intracellular adhesion molecule (ICAM-1) 15 μl/ml (Santacruz Biotechnology); vascular endothelial cell adhesion molecule (VCAM-1) 5 μl/ml (Santacruz Biotechnology); fibronectin (FN) 2.5 μl/ml (Sigma), phospho-S536 p65 1:50 (Abcam); phospho-AKT 1:200 (Abcam); MAC2 1:10,000 (CedarLane Laboratories); and phosphoS552-RelB 1:50 (Abcam). Sections were incubated overnight at 4°C and visualized with Vectastain Elite Kit (Vector Labs) with diaminobenzidine (DAB; Deko Corp). Sections were counterstained with Hematoxylin and exposed to 1 min of Bluing solution for ICAM-1, VCAM-1, FN, p-AKT, MAC2, and p- RelB and with Fast Green for p-p65. Positive (ApoE ^-/-^ mice fed with a high fat diet) and negative controls validated the staining for each of the inflammatory markers (data not shown).

### Imaging and analyses

Images were acquired using the 10X and 40X objectives on an Olympus BX51 microscope equipped with an Olympus DP70 digital camera using the ImagePro Plus 7.0 software program in the Academic Computing Health Sciences Center at the University of Virginia. 7-8 images were acquired each for the greater and lesser curvature per animal. Image J software was used to quantify the intensity of endothelial staining between strains in lesser curvature (atheroprone) and in greater curvature (atheroprotective).

**Figure 1:**
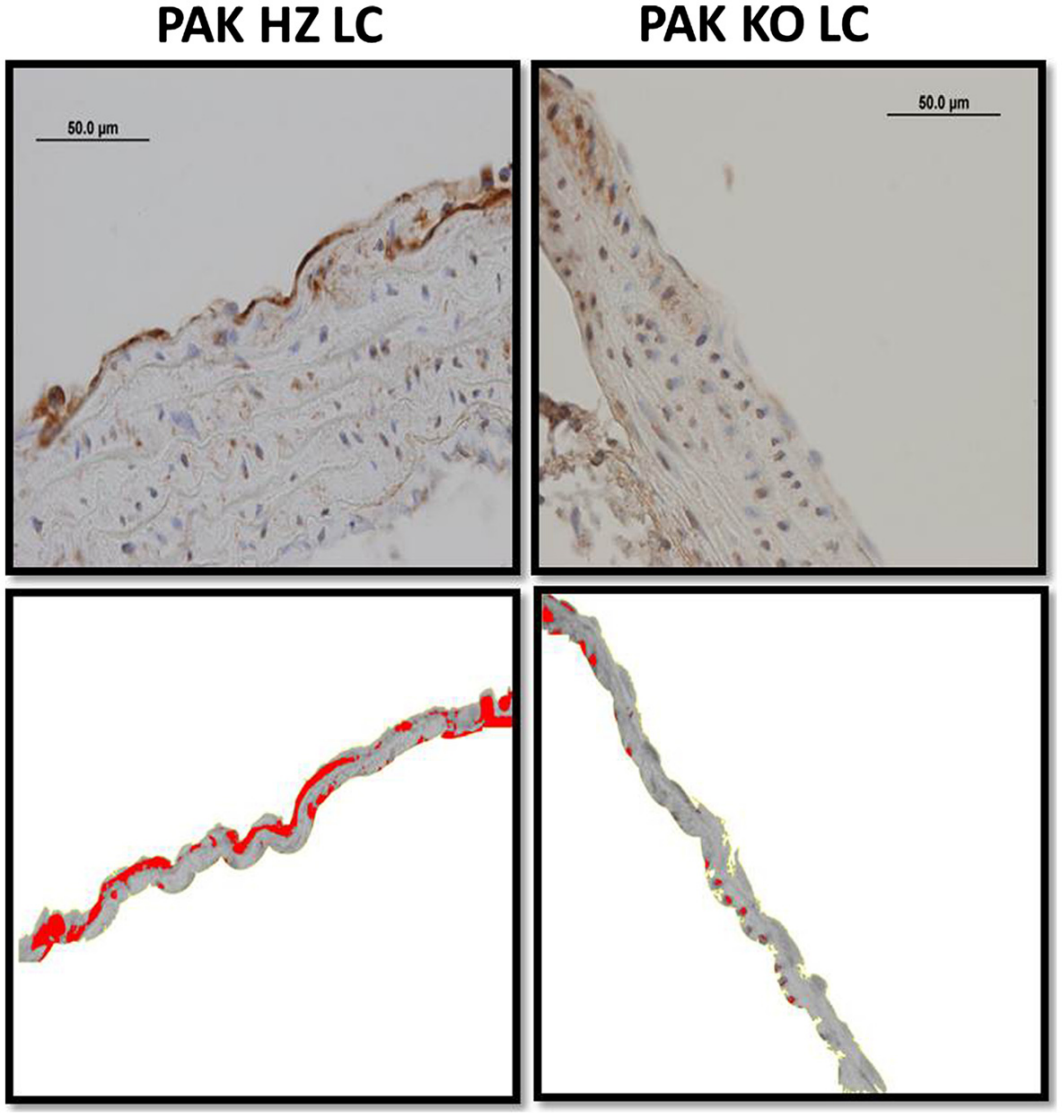
**Automated image analysis.** The Image J protocol is illustrated using fibronectin staining in the lesser curvature of PAK HZ and PAK KO mice. Minimal threshold intensity is selected such that only the endothelial cell stained regions are positive. The inner 90 pixels are isolated as the endothelial layer. Consistent area selection across sections gives the mean integrated density in endothelial cells as a function of area and intensity. Image J analysis cartoon of DAB stained regions (red) in the lesser curvature (LC) of PAK heterozygote (HZ) [left] and knockout (KO) mice [right].

Staining was then quantified using an automated procedure. We first determined that the intima (from the endothelium to the first lamina) was consistently 90 pixels wide. We next manually determined the threshold intensity that corresponds to the minimal detectable intensity in that region that excluded non-specific staining. To achieve consistency, the thresholds for each of the images (4-6 images per sample) were averaged and the averaged threshold was used for each animal. This correlation of constant area and averaged threshold gave us integrated density (product of the area and mean gray value that reflected each pixel staining). Analysis was carried out with consistent area selection across all markers, sections and strains. The selected areas and thresholds are illustrated in Figure 1. This procedure gave us consistent intensity data between samples.

## Results

**Figure 2:**
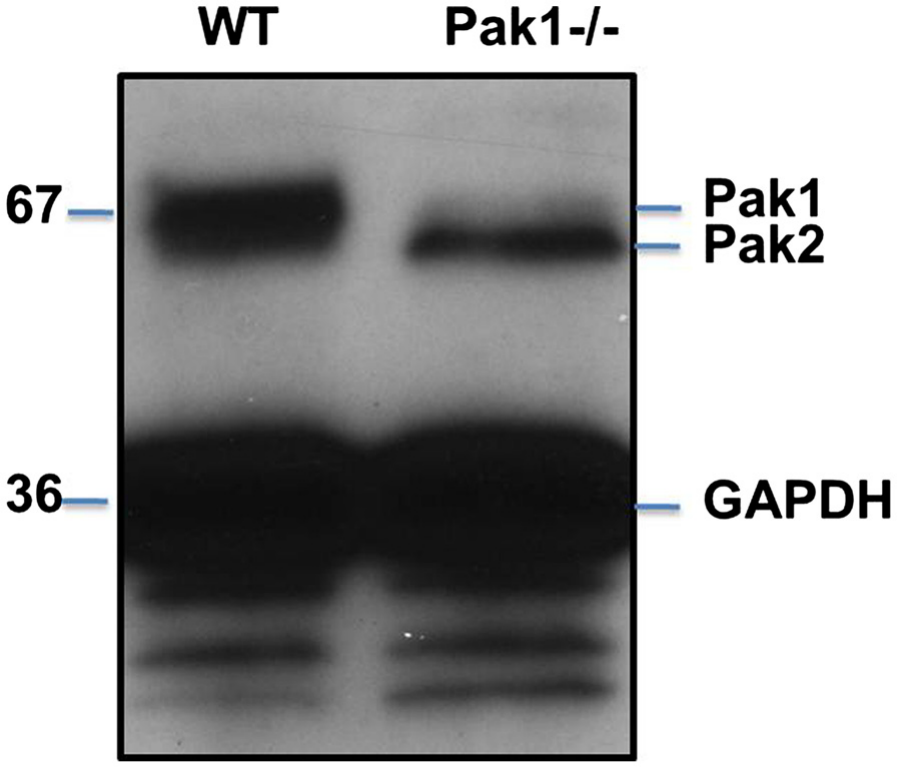
**PAK1 in mouse endothelial cells.** Western Blotting showing PAK1 expression in mouse endothelial cell lysate. Recombinant PAK1 protein was used as a positive control.

Western blotting of lysate from isolated primary mouse endothelial cells (ECs) with an isoform-specific antibody showed PAK1 expression in these cells (Figure 2). We therefore investigated its role in the inflammatory activation of the endothelium in WT and PAK1-/- mice. We compared the lesser (inner) curvature of the aortic arch, a site of flow disturbance, to the atherosclerosis-resistant greater (outer) curvature where flow is laminar. We examined arteries from 10-12 week old PAK KO, PAK HZ, and WT C57BL/6 mice using a suite of markers for proteins that have been implicated in atherosclerosis or flow signaling. The expression of the selected markers was comparable between PAK HZ and C57BL/6 (data not shown), thus, only the PAK HZ mice were used for detailed comparison with the PAK1 KO mice.

**Figure 3:**
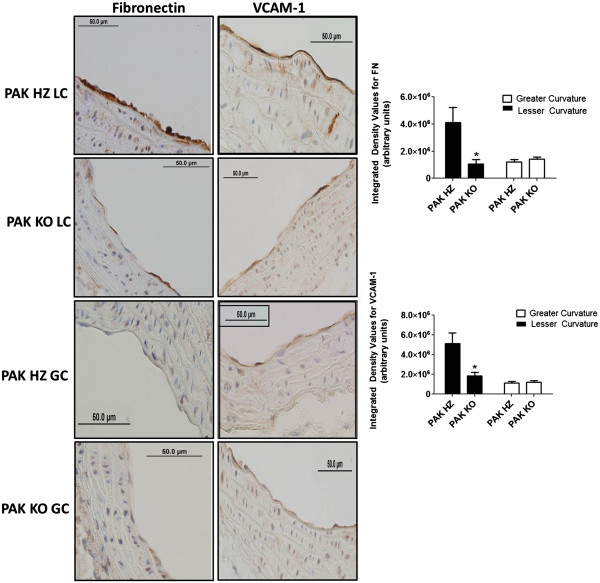
**Inflammatory markers FN and VCAM-1 in the lesser and greater curvature of PAK HZ and PAK KO mice.** Fibronectin (FN) [left panel] and VCAM-1 [right panel] were stained and imaged at 40X magnification in the lesser and greater curvatures of the PAK heterozygote (HZ) and knockout (KO) mice [Panels top to bottom]. Each section was imaged at 5 different locations within a curvature zone to cover the entire length of the greater (GC) and lesser curvature (LC), respectively. The bar graphs below each panel show the integrated density between the two strains in the region of the greater and lesser curvatures. Sample size was n = 6 for PAK HZ and n = 7 for PAK KO. “*” represents significance <0.05 as analyzed by independent Student t-test.

**Figure 4:**
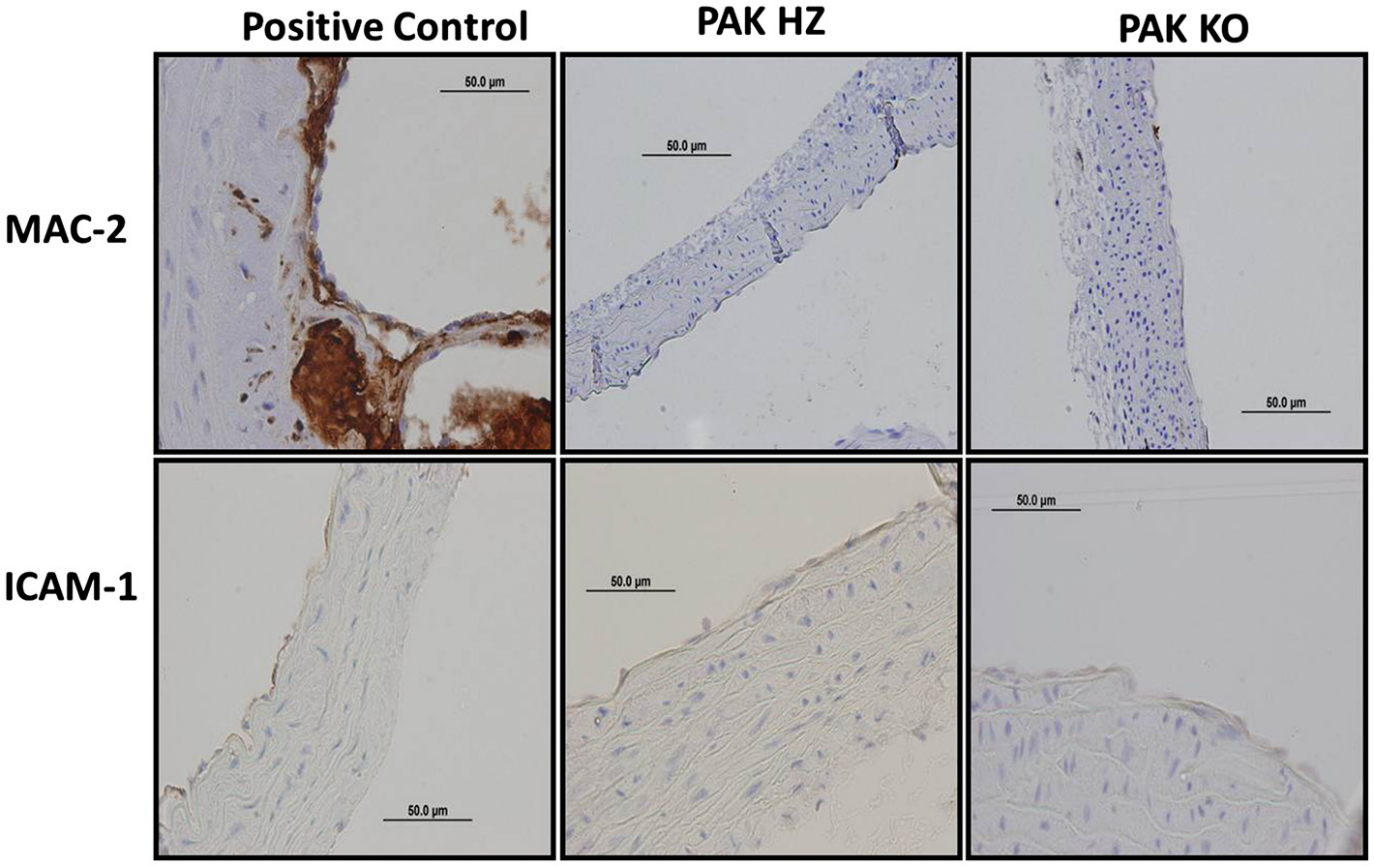
**Mac2 and ICAM-1 staining in the lesser curvature arch of PAK HZ and PAK KO mice.** The *top panel* shows Mac2 staining (from left to right) in the lesser curvature of ApoE null mice (positive control), PAK HZ, and PAK KO mice. In a similar representation, ICAM-1 staining is demonstrated in the lower panel. N = 5 for PAK HZ and PAK KO mice.

We first stained for fibronectin (FN) as a marker for matrix remodeling. FN localized specifically to the lesser curvature as expected, but was strongly decreased in PAK1 KO mice (Figure 3*left panel*). We also examined expression of the leukocyte adhesion receptors VCAM-1 and ICAM-1 as markers for immune activation of the endothelium, and Mac2 to identify infiltrating monocytes or macrophages. VCAM-1, which also specifically localized to the lesser curvature relative to the greater curvature, was decreased in the PAK KO compared to PAK HZ mice (Figure 3*right panel*). Neither ICAM-1 (Figure 4*lower panel*) nor Mac2 (Figure 4*top panel*) were detected in any of the mice. These results are consistent with the age of the mice (10-12 weeks), since inflammation is low in young C57Bl/6J mice fed on a chow diet, though it increases with age (unpublished data).

**Figure 5:**
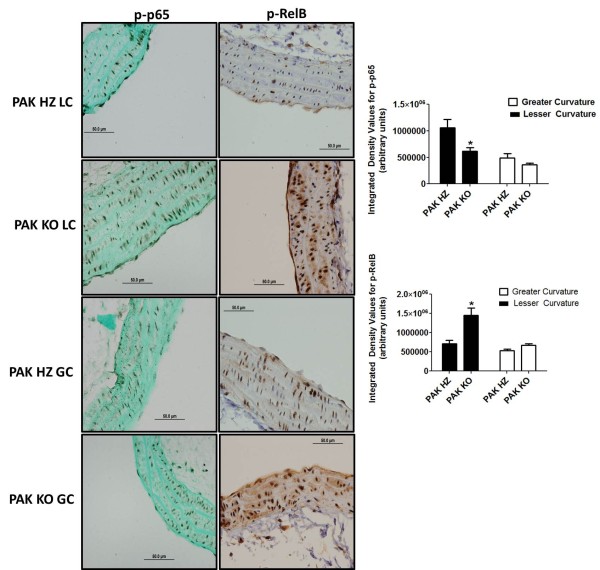
**Nuclear Factor (NF) – κB staining.** DAB staining and image analysis reflecting p-p65 endothelial staining (left panel) and p-RelB staining (right panel) in PAK HZ and PAK KO mice. Each protein staining is seen in the lesser curvature (LC) and greater curvature (GC) between the two strains. ‘*’ indicates significance p < 0.05 between the two strains of mice. N = 6 in PAK HZ and n = 7 for PAK KO mice.

**Figure 6:**
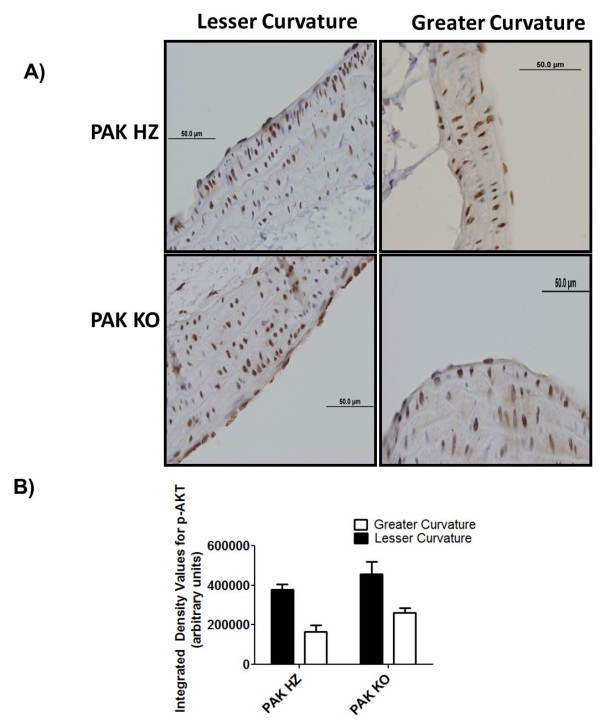
**Akt activation.A**) DAB staining revealed p-AKT staining in the lesser curvature (LC) of PAK HZ (Left panel) and PAK KO (Right panel). **B**) Intensity analysis of DAB staining between PAK HZ and KO knockout (KO) mice. N = 5 for each strain.

To detect activation of a critical pro-inflammatory signaling pathway, we assayed activation of classical NF-κB by staining for the RelA (p65) subunit phosphorylated on an activating site, S536 (p-p65), which was previously shown to be stimulated by fluid shear stress [19]. Staining localized specifically to the lesser curvature, as expected, and was significantly decreased in PAK1-/- vessels (Figure 5). Interestingly, staining for the alternative NF-κB subunit RelB with an antibody against an activating phosphorylation site (pRelB), which was also elevated in the lesser relative to the greater curvature, was much higher in PAK1-/- mice than heterozygotes (Figure 5). Staining for p-AKT did not reveal any difference between the two strains (Figure 6). However, the phosphorylation was different between the lesser and greater curvatures within the respective strains.

## Discussion

In vitro studies identified a critical role for PAK family kinases in the inflammatory activation of endothelial cells by fluid shear stress but in vivo evidence is minimal and the isoforms involved were not identified. The current study was designed to explore the in vivo importance of PAK in this process, focusing on PAK1. Our results showed that expression or activation of FN, VCAM-1, and classical NF-κB (RelA) were reduced in aortas from PAK1-null mice. Decreased inflammatory activation in PAK1-null mice fits well with in vitro findings from our laboratory that PAK promotes inflammation in response to fluid shear stress [12-14]. These data also highlight the importance of the PAK1 isoform in these pathways. By contrast, PAK1 did not detectably affect p-AKT, consistent with Akt’s role as a direct mediator of flow signaling rather than an inflammatory mediator. Levels of Mac-1 and ICAM-1 were very low, consistent with the young age of the mice and the low level of inflammation at this stage.

Interestingly, RelB, a component of the alternative NF- κB pathway, was also activated in the lesser curvature of the arch, but its activation greatly increased in the absence of PAK1 expression. RelB-/- mice exhibit multi-organ inflammation, suggesting that this subunit is mainly immune-suppressive [20]. If it performs a similar function in the endothelium, then elevated RelB activity in PAK1-/- aortas is consistent with reduced inflammation in these mice. However, RelB has not been studied in endothelial cells, thus, its functional role remains to be investigated.

Importantly, WT C57Bl6 mice do not develop atherosclerosis in the absence of other stimuli such as hypercholesterolemia [6]. Indeed, monocyte/macrophage accumulation was nearly undetectable. Thus, the mild inflammatory activation seen in this region is most likely biomechanical. These results therefore suggest that PAK1 plays a significant pro-inflammatory role in an atherosclerosis-prone arterial region subject to disturbed flow.

## Conclusions

In conclusion, these results demonstrate a role for PAK1 in promoting inflammation at sites of disturbed flow in vivo. This effect may be exerted via NF-κB activation, gene expression and fibronectin deposition. Further studies in hypercholesterolemic ApoE null or LDLR null mice are required to determine whether these early changes will translate into reduced atherosclerosis.

## Competing Interests

The authors declare that they have no competing interests.

## Authors Contribution Section

KAJ carried out the cell culture work, tissue dissection from the mice, staining, imaging, analyses, graph plotting, and drafted the manuscript. PD participated in imaging of tissue sections and its analyses. JC provided all the mice used in the study. JS carried out the processing and sectioning of the tissues and the antibody dilution series for all the immunohistochemistry stains. MAS participated in study design and coordination and helped to draft the manuscript. All authors read and approved the final manuscript.

## Pre-publication history

The pre-publication history for this paper can be accessed here:

http://www.biomedcentral.com/1471-2261/12/55/prepub
